# Psychological assessment in patients affected by trigeminal neuralgia. A systematic review

**DOI:** 10.1007/s10143-025-03556-4

**Published:** 2025-05-13

**Authors:** Renata Martinelli, Sofia Vannuccini, Benedetta Burattini, Quintino Giorgio D’Alessandris, Manuela D’Ercole, Alessandro Izzo, Daniela P. R. Chieffo, Francesco Doglietto, Nicola Montano

**Affiliations:** 1 Department of Neurosurgery, Fondazione Policlinico Universitario Agostino Gemelli IRCCS, largo A. Gemelli, 8, Rome, 00168 Italy; 2 Department of Neuroscience, Università Cattolica del Sacro Cuore, Largo F. Vito, 1, Rome, 00168 Italy

**Keywords:** Trigeminal neuralgia, Psychological assessment, Microvascular decompression, Sleep disorder, Depression, Anxiety

## Abstract

The aim of the present study was to conduct a systematic review regarding the presence and type of psychological comorbidities in patients with trigeminal neuralgia and to determine the potential impact of surgical treatments on these conditions. We reviewed the literature following PRISMA guidelines to identify and critically examine relevant studies. The review question was formulated according to the PICO framework as follows: “For patients affected by trigeminal neuralgia (P) undergoing neuropsychological assessments (I) and potentially undergoing reassessment after surgical treatment (C), is there a correlation between psychological issues and trigeminal neuralgia, and does the treatment of trigeminal neuralgia improve psychological well-being (O)?”. The literature search yielded a total of 316 results. After removing duplicates (*n* = 73), 243 papers were screened. Following title and abstract screening, 222 records were excluded. Ultimately, 11 studies were deemed relevant to the research purpose. To the best of our knowledge, this is the first systematic review highlighting the significant psychological burden of trigeminal neuralgia, including higher risks of sleep, depressive, and anxiety disorders. Surgical treatments effectively alleviate both pain and psychological symptoms, and multidisciplinary approaches combining psychological support and neuro-rehabilitation with medical or surgical care yield better outcomes. Standardizing psychological assessment and treatment methodologies is crucial for optimizing trigeminal neuralgia management. Clinical trial number: Not applicable.

## Introduction

Trigeminal neuralgia (TN) is a neuropathic condition characterized by severe facial pain occurring along the distribution of the trigeminal nerve. This debilitating disorder significantly impacts the quality of life of affected individuals, leading to profound physical and psychosocial burdens [23, 32]. TN is typically described as a sudden, sharp pain resembling a stab or electric shock, localized to one or more branches of the trigeminal nerve. The clinical presentation of TN can be broadly categorized into two types: classical TN, which manifests as episodic pain with pain-free intervals (now referred to as TN with pure paroxysmal pain), and atypical TN, characterized by continuous or sub-continuous pain (now termed TN with continuous pain) [6].

Despite extensive research, the exact etiology of TN remains elusive. The role of systemic inflammation and of specific biomarkers indicative of localized nerve damage, particularly in cases involving neurovascular conflict, has been increasingly recognized in recent studies [22]. Neurovascular conflict, often identifiable via preoperative imaging, is the most common cause of TN, and microvascular decompression (MVD) is considered the gold standard treatment in such cases [1, 32]. In addition, despite surgical treatment becoming increasingly individualized, thanks in part to advancements in intraoperative neuromonitoring techniques [13, 19], a substantial subset of patients lacks clear evidence of neurovascular compression, leaving the optimal management strategy undefined [20].

Recent evidence has highlighted the interplay between TN and psychiatric conditions, emphasizing the bidirectional relationship between chronic pain and mental health. While the association between migraine and psychiatric disorders has been extensively studied, only recently has attention shifted to the psychological dimensions of TN [9, 12, 31]. Patients with TN frequently experience comorbid depression, anxiety, and sleep disturbances, all of which exacerbate their overall disability. This is particularly relevant since TN predominantly affects individuals in their working years or those at a stage of life where independence in activities of daily living (ADLs) is critical [8]. The inability to perform even routine tasks such as chewing or brushing teeth further compounds the social and psychological impact of TN, contributing to a significant disability.

Chronic TN, if inadequately managed, has been associated with the development of psychiatric morbidities, which may in turn predict poorer treatment outcomes. Newly diagnosed psychiatric disorders in these patients could serve as markers for chronic pain severity and treatment resistance, necessitating a multidisciplinary approach to care.

This study aims to review systematically the existing literature, in order to evaluate the presence of psychological comorbidities in patients with TN, and potentially to determine the impact of surgical treatments on these conditions by recording variations in neuropsychological assessments before and after treatment.

## Methods

This systematic review adhered to established guidelines, such as PRISMA (Preferred Reporting Items for Systematic Reviews and Meta-Analyses), for identifying and critically evaluating relevant studies. All procedures followed the protocols outlined in the Cochrane Handbook of Systematic Reviews and Meta-analysis of Interventions (version 6.3) [7, 21]. The review question was structured using the PICO (Population, Intervention, Comparison, Outcomes) framework: “For patients affected by trigeminal neuralgia (P) undergoing neuropsychological assessments (I) and potentially undergoing reassessment after surgical treatment (C), is there a correlation between psychological issues and trigeminal neuralgia, and does the treatment of trigeminal neuralgia improve psychological well-being (O)?” [24]. Electronic databases (PubMed, Scopus) were search using comprehensive search terms: “(Trigeminal neuralgia) AND (Psychology)”.

The latest search was conducted in November 2024. Two authors (R.M. and S.V.) independently conducted the abstract screening for eligibility. Any discordance was solved by consensus with a third, senior author (N.M.). Studies meeting the following criteria were included: studies that reported psychological assessment in patients affected by TN with appropriate scales, comparing evaluation between pre and postoperative period, or investigating the association between TN and psychological issues. Exclusion criteria comprised: studies published in languages other than English, systematic reviews, case reports, studies including less than 3 patients affected by TN, letters to the editor. Extracted data encompassed study characteristics, patient demographics, surgical details, type of TN, type of psychological assessment, pain relief and recurrences, and outcomes. The risk of bias was assessed using the ROBINS-I (Risk of Bias in Non-randomized Studies - of Interventions) assessment tool.

## Results

**Fig. 1:**
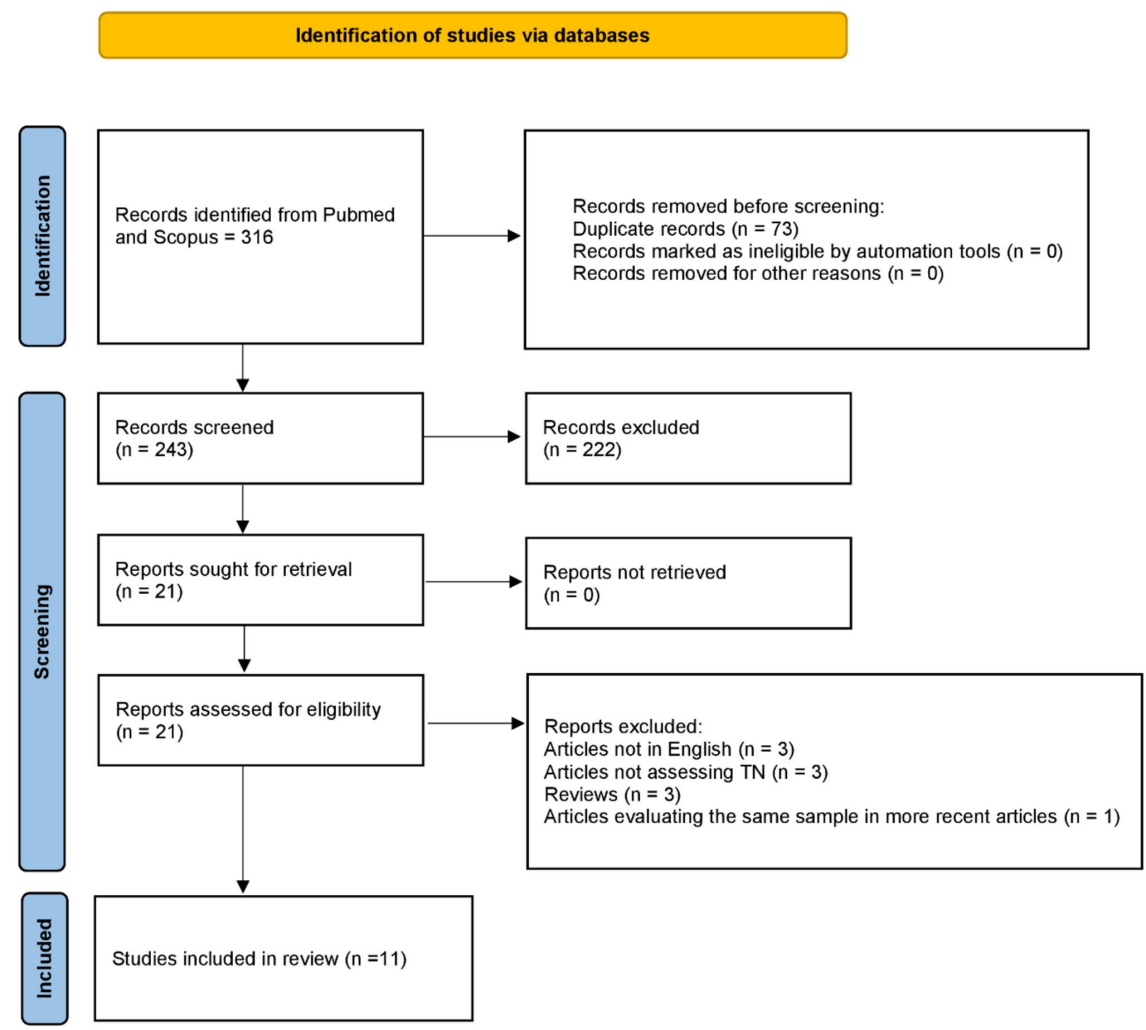
PRISMA flow diagram

**Fig. 2:**
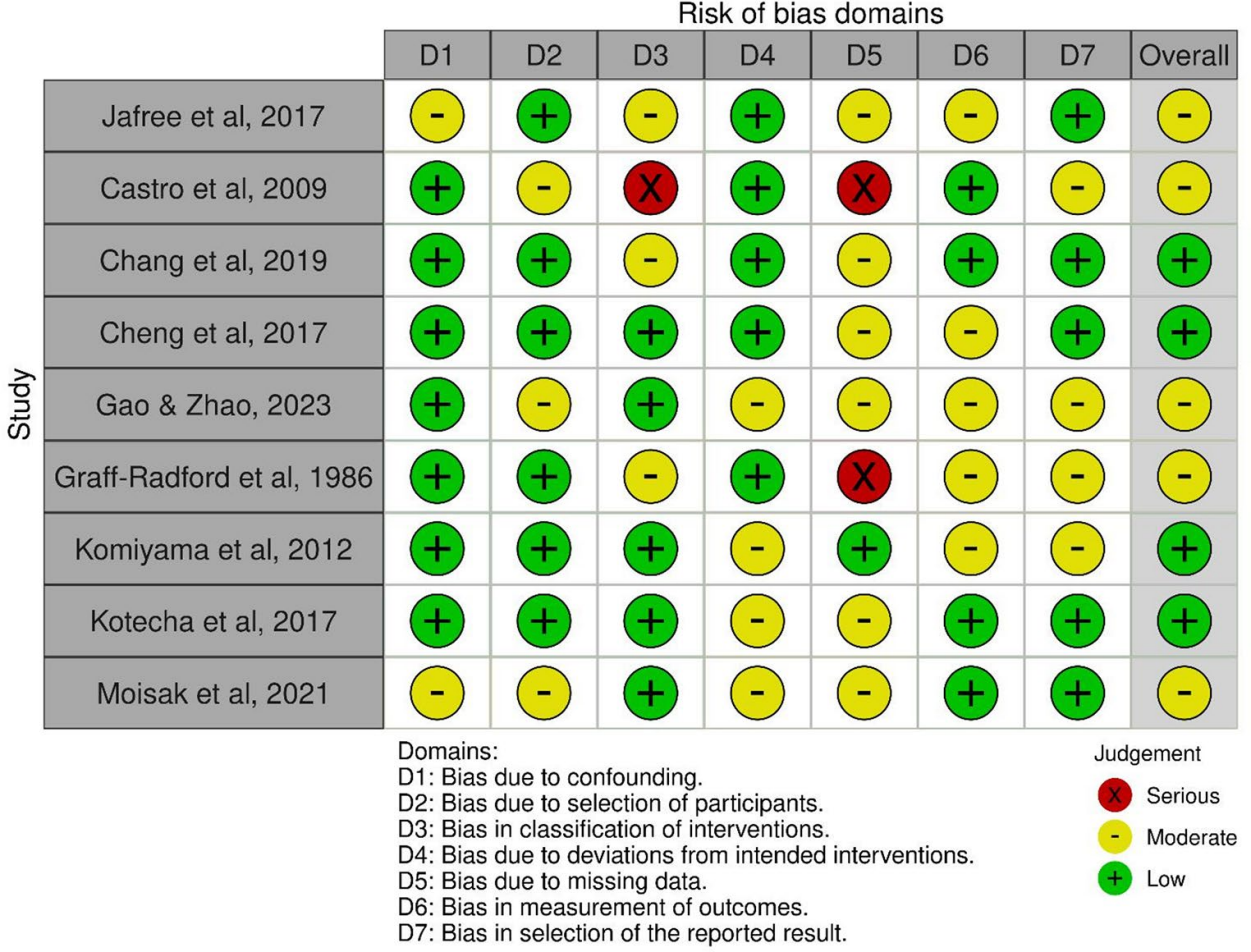
Risk of bias assessment

The search of the literature yielded 316 results. Duplicate records were then removed (*n* = 73). A total of 243 papers were screened, and 222 records were excluded via title and abstract screening. Of the 21 fulltexts assessed, 10 were found not to be relevant to our research question, thus 11 papers were included in the present review (Fig. 1). From the analysis of the 11 selected fulltexts, including 6 retrospective studies and 5 prospective studies (Table 1), it was not possible to extract data to perform a quantitative analysis. Nonetheless, a qualitative evaluation was made leading to the identification of the following five main topics: (1) psychological comorbidities in TN, (2) psychological outcomes of surgical interventions, (3) alternative treatments and psychological support, (4) long-term psychosocial dynamics related to chronic trigeminal pain, (5) neuro-rehabilitation and psychological interventions. The results of risk of bias assessment are reported in Fig. 2.

**Table 1:** Studies included in this systematic review

Author, year	Type of study	Follow-up (months)
Castro et al., 2009 [2]	retrospective	NA
Chang et al., 2019 [4]	retrospective	6
Cheng et al., 2017 [5]	prospective	6
Gao et al., 2023 [10]	prospective	3
Graff-Radford et al., 1986 [11]	retrospective	NA
Jafree et al., 2018 [14]	retrospective	6
Komiyama et al., 2012 [16]	retrospective	NA
Kotecha et al., 2017 [17]	prospective	13
Moisak et al., 2021 [18]	prospective	14
Wang et al., 2024 [29]	prospective	6
Wu et al., 2015 [30]	retrospective	NA

NA = not available

## Discussion

The aim of this systematic review was to evaluate the presence and the type of psychological comorbidities in patients affected by TN and to determine, when possible, the impact of surgical treatments for TN on these comorbidities. To the best of our knowledge, this is the first systematic review on the issue. The five main topics identified are discussed in detail in the following paragraphs.

### Psychological comorbidities in trigeminal neuralgia

Wu et al. [30] conducted a study comparing the prevalence of psychiatric disorders in patients with TN and in the general population. Their findings revealed that TN patients are at a significantly higher risk of developing sleep disorders, depressive disorders, and anxiety disorders compared to the general population (*P* < 0.001). No significant differences were observed between the groups regarding the onset of other psychiatric conditions, such as bipolar disorder or schizophrenia.

These treatable psychiatric disorders significantly affect the quality of life of TN patients,

**Table 2:** Studies evaluating the psychological comorbidities in TN

Author, year	*N* patients	Psychological assessment	Conclusions
Wu et al., 2015 [30]	TN = 3273 Control = 13,092	ICD-9-CM	TN might increase the risk of subsequent newly diagnosed depressive disorder, anxiety disorder, and sleep disorder, but not schizophrenia or bipolar disorder.
Komiyama et al., 2012 [16]	BMS = 282 TN = 83	RDC-TMD (Axis II questionnaire)	Pain intensity is not directly correlated with psychological impact. Continuous mild-to-moderate pain has negative psychosocial effects on well-being.

BMS = Burning mouth syndrome; ICD-9-CM = International Classification of Diseases, Ninth Revision, Consensus Modified; RDC-TMD: Research Diagnostic Criteria for Temporomandibular Disorders; TN = Trigeminal neuralgia

emphasizing the importance of healthcare providers being vigilant about the potential development of depressive, anxious, or sleep disorders in this population (Table 2).

Komiyama et al. [16] compared the perceived pain intensity and psychosocial characteristics between classical TN and Burning Mouth Syndrome (BMS), using the Research Diagnostic Criteria for Temporomandibular Disorders (RDC-TMD) Axis II questionnaire [25]. The results indicated that, although pain intensity was more severe in classical TN than in BMS, the psychosocial impact of BMS was similar or greater due to somatization. Atypical TN patients were excluded. These findings suggest that pain intensity is not directly correlated with psychological impact, as mild to moderate chronic pain also exerts negative psychosocial effects (Table 2).

### Psychological outcomes of TN surgical interventions

Moisak et al. [18] demonstrated a significant psychological improvement in anxiety and depression among TN patients following surgical interventions, highlighting the dual benefit of pain relief and psychological recovery. Preoperatively, elevated scores on anxiety and depression scales (e.g., Hospital Anxiety and Depression Scale, HADS) were noted [15], although most patients did not meet the diagnostic threshold for Depressive or Anxiety Disorders. Postoperatively, anxiety symptoms initially increased due to fear of recurrent severe pain, but tended to resolve after complete pain relief from MVD. Despite surgical success, some patients experienced behaviors such as symptom exaggeration or catastrophizing, leading to an underestimation of the efficacy of surgery, and suggesting a need for psychological support during social reintegration post-surgery. Notably, although the anxiety level of patients with chronic, atypical pain was comparable to patients experiencing typical symptoms, the risk of suicidal ideation in the atypical TN patients was significantly higher than in those with classical TN (Tables 3 and 4).

Cheng et al. [5] further confirmed that TN patients exhibit higher anxiety and depression levels than the general population. Their follow-up at 3 and 6 months post-surgery using the Beck Anxiety Inventory (BAI) and Beck Depression Inventory-II (BDI) revealed a reduction in symptoms, suggesting that effective pain relief from MVD significantly alleviates pain-induced psychiatric symptoms [15, 27] (Tables 3 and 4).

Jafree et al. [14] assessed long-term psychological outcomes in TN patients 5 years post-surgery, finding greater anxiety and depression in patients treated with partial sensory rhizotomy (PSR) compared to MVD, presumably due to a higher incidence of postoperative complications, such as facial sensory loss, in PSR. Nonetheless, quality of life in post-surgical TN patients remained lower than in age-matched controls, underscoring the long-term nature of TN as a chronic condition (Tables 3 and 4).

Chang et al. [4] demonstrated a strong correlation between anxiety, depression and TN, with these psychiatric symptoms positively linked to pain severity and duration. Their study highlighted the importance of psychiatric intervention, as untreated anxiety and depression negatively influenced surgical outcomes (Tables 3 and 4).

Kotecha et al. [17] evaluated the efficacy of stereotactic radiosurgery (SRS) on psychological comorbidities in TN, finding an improvement of both pain perception and quality of life, alongside a reduction in depressive symptoms, without significant differences between patients affected by classical or atypical TN (Tables 3 and 4).

**Table 3:** Studies reporting the psychological outcomes of TN surgical interventions

Author, year	*N* Patients	Psychological assessment	Conclusions
Moisak et al., 2021 [18]	56	HADS, GDS	Psychological assessments help guide treatment decisions in TN patients and clarify intervention timing. Regardless of outcomes, patients require psychological support for social adaptation to changes in their status. The anxiety level did not differ between patients affected by classical and atypical TN, but the risk of suicidal ideation was significantly higher in the atypical TN patients.
Cheng et al., 2017 [5]	128	BDI, BAI	Depression and anxiety are prevalent in patients with idiopathic TN. Female gender, high pain intensity and ineffective medicine treatment are risk factors. MVD not only provides high pain-relief rate, but also leads to significant improvements in the depression and anxiety symptoms.
Jafree et al., 2018 [14]	230	HADS	QOL 5 years after MVD or PSR is poorer than in the general population and it is associated with postoperative complications, which are commoner after PSR than MVD. Therefore, PSR is associated to greater anxiety and depression than MVD.
Chang et al.; 2019 [4]	TN = 45 Control = 61	HDRS; HARS	TN patients were associated with remarkably more elevated HDRS and HARS scores compared with controls, suggesting the association of depression and anxiety with TN, which may also affect the outcome of patients undergoing MVD
Kotecha et al., 2017 [17]	50	EQ-5D, PHQ-9	Patients with TN treated with SRS reported significant improvements in multiple QOL measures, with the therapeutic benefit strongly driven by improvements in pain and discomfort and in self-care, along with lower rates of depression. No significant difference was found between patients affected by classical and atypical TN.

BAI = Beck Anxiety Inventory; BDI = Back Depression Inventory; EQ-5D: European Quality of Life 5 Dimensions; HADS = Hospital Anxiety and Depression Scale; HDRS = Hamilton Depression Rating Scale; MVD = Microvascular Decompression; PHQ-9 = Patient Health Questionnaire-9; QOL = Quality of Life; SRS = Stereotactic Radiosurgery; TN = Trigeminal neuralgia

**Table 4:** Detail on preoperative and postoperative assessment of depression and axiety scores

Author, year	*N*. Patients	Psychological assessment	Pre-treatment score (mean)	Post-treatment score (mean)	Outcome
Moisak et al., 2021 [18]	56	HADS GDS (25/56)	HADS = 6.2 GDS = 5.5	HADS = 6.6 GDS = NA	Non-significant increase of anxiety level after treatment, due to the fear of pain recurrence.
Cheng et al., 2017 [5]	128	BDI BAI	BDI = 19.3 BAI = 38.5	BDI = 6.8 BAI = 25.6	Significant improvements both in depression and in anxiety.
Jafree et al., 2018 [14]	230	HADS	NA	NA	Patients were over 3 times more likely to have anxiety after PSR than after MVD.
Chang et al., 2019 [4]	TN = 45 Control = 61	HDRS HARS	HDRS = 10.9 HARS = 11.2	HDRS pp group = 16.9 pf group = 9.6 HARS pp group = 15.5 pf group = 10.5	Depression and anxiety worsened in persistent pain group.
Kotecha et al., 2017 [17]	50	EQ-5D PHQ-9	EQ-5D = 0.525 PHQ-9 = 9	EQ-5D = 0.690 PHQ-9 = 6	QOL improved after SRS.

BAI = Beck Anxiety Inventory; BDI = Back Depression Inventory; EQ-5D: European Quality of Life 5 Dimensions; GDS, Geriatric Depression Scale; HADS = Hospital Anxiety and Depression Scale; HDRS = Hamilton Depression Rating Scale; MVD = Microvascular Decompression; PHQ-9 = Patient Health Questionnaire-9; pf = pain-free; pp = persistent pain; QOL = Quality of Life; SRS = Stereotactic Radiosurgery; TN = Trigeminal neuralgia

### Non-surgical interventions and psychological support

Evidence underscores the importance of psychological treatment for TN patients both pre- and postoperatively, as psychological profiles influence surgical outcomes. Gao et al. [10] demonstrated the effectiveness of a humanistic nursing care model in improving psychological well-being and postoperative recovery, emphasizing the benefits of patient-centered care, emotional support, and tailored education in reducing psychological burden and enhancing overall recovery (Table 5).

### Long-term psychosocial dynamics related to chronic trigeminal pain

Graff-Radford et al. [11] retrospectively examined the psychological status of TN patients using the MMPI (Minnesota Multiphasic Personality Inventory) [26], revealing a complex interplay between psychological adaptation and pain perception, where psychological distress exacerbated the pain.

**Table 5:** Study reporting about non-surgical interventions and psychological support in TN patients

Author, year	*N* Patients	Psychological assessment	Conclusions
Gao et al., 2023 [10]	166 Observation group = 88 Control group = 78	SAS, SDS	Implementing a humanistic care-based nursing model effectively enhances the psychological well-being and recovery quality of TN outpatients attending pain clinics.

SAS = Self-rating Anxiety Scale; SDS = Self-rating Depression Scale

**Table 6:** Studies about the long-term psychosocial dynamics related to chronic trigeminal pain

Author, year	Year	*N* Patients	Psychological assessment	Conclusions
Graff-Radford et al., 1986 [11]	1986	TN = 15, PHN-late = 15 PHN-early = 6	MPQ, MMPI, CIPI	Greater psychological dysfunction and disability are associated with the continuous unrelenting pain of PHN as compared with the sharp intermittent pain of TN, independent of overall intensity.
Castro et al., 2009 [2]	2009	TN = 15 TMD = 15	HADS	Patients who were hospitalized to treat their orofacial pains were more anxious and had more expectations about the treatment when compared to other groups. Patients affected by chronic facial pain need psychological support to cope with high expectations towards surgical treatment.

CIPI = Chronic Illness Problem Inventory; MMI = Modified Mercalli Intensity Scale; MPQ = McGill Pain Questionnaire; PHN = Postherpetic Neuralgia; TMD = Temporomandibular Disorder; TN = Trigeminal Neuralgia

**Table 7:** Study reporting about neurorehabilitation and psychological interventions in TN patients

Author, year	Number of Patients	Psychological assessment	Conclusions
Wang et al., 2024 [29]	120 Observation group = 60 Control group = 60	VAS, SAS, SDS	Comprehensive rehabilitation intervention can effectively reduce the degree of post-operative pain in patients with trigeminal neuralgia, help to regulate their psychological state and reduce the occurrence of complications.

SAS = Self-rating Anxiety Scale; SDS = Self-rating Depression Scale; VAS = Visual Analogue Scale

experience (Table 6). Similarly, Castro et al. [2, 3] highlighted the emotional toll of chronic orofacial pain and the need for coping strategies and psychological interventions. Chronic pain in TN and TMD patients had more profound psychological repercussions than in other conditions, impairing quality of life, limiting workability, and eliciting poor family support, which collectively contributed to distress, anxiety, and depression [2, 3].

### Neurorehabilitation and psychological interventions

Wang et al. [29] explored the role of neurorehabilitation in postoperative psychological recovery, employing the Self-Rating Anxiety Scale (SAS) and Self-Rating Depression Scale (SDS) at multiple intervals pre- and post-surgery [28]. Psychological suggestion therapy, combining psychological intervention with suggestion techniques, positively influenced patients’ understanding and management of their clinical and psychological conditions. This comprehensive rehabilitative approach effectively reduced postoperative pain scores, SAS, and SDS scores, minimized complications, and consolidated therapeutic outcomes, confirming its efficacy in enhancing the emotional well-being and recovery of TN patients (Table 7).

### Limitations

Despite the shared insights, the studies differ in their focus on surgical versus non-surgical interventions and in the extent of psychological assessments performed. While some studies provided longitudinal data with detailed follow-ups, others relied on cross-sectional designs or limited psychological metrics. These methodological variations may influence the reported prevalence of psychiatric comorbidities and the perceived effectiveness of treatments.

Cultural and methodological differences also emerged as notable factors affecting the generalizability of findings. Variations in healthcare access, cultural perceptions of pain, and the availability of psychological support may contribute to discrepancies in prevalence rates and treatment outcomes. For instance, studies conducted in regions with robust healthcare systems and integrated mental health services reported more comprehensive care and better recovery trajectories than those performed in areas with limited resources.

Other significant limitations in the literature analysis include: the inconsistent differentiation between types of TN (classical and atypical); the absence of evaluation of the potential psychological impact of pain recurrence; and the insufficient assessment of the correlation between perioperative changes in pain intensity (e.g., using BNI scale) and perioperative variations in psychological scales.

## Conclusions

This systematic review underscores the profound psychological burden of patients with TN. Anxiety, depression and, to a lesser extent, sleep disorders and suicidal ideation emerged as the main issues associated with TN.

Notably, neurosurgery has shown positive results both for alleviating pain and psychological symptoms. The adoption of a multidisciplinary approach is of paramount importance: pain treatment should be combined to psychological support and neuro-rehabilitation in order to obtain better outcomes. Future research should aim to standardize methodologies for assessing psychological comorbidities and treatment outcomes in TN patients, in order to optimize individualized treatment strategies for these patients.

## Data Availability

No datasets were generated or analysed during the current study.

## Abbreviations

ADLs: Activities of Daily Living
BAI: Beck Anxiety Inventory
BDI: Beck Depression Inventory-II
BMS: Burning Mouth Syndrome
BNI: Barrow Neurological Institute (specific reference to pain scale in TN)
CIPI: Chronic Illness Problem Inventory
EQ-5D: European Quality of Life 5 Dimensions
HADS: Hospital Anxiety and Depression Scale
HARS: Hamilton Anxiety Rating Scale
HDRS: Hamilton Depression Rating Scale
ICD-9: International Classification of Diseases, Ninth Revision
MMI: Modified Mercalli Intensity Scale
MMPI: Minnesota Multiphasic Personality Inventory
MPQ: McGill Pain Questionnaire
MVD: Microvascular Decompression
PHN: Postherpetic Neuralgia
PHQ-9: Patient Health Questionnaire-9
PICO: Population, Intervention, Comparison, Outcomes
PRISMA: Preferred Reporting Items for Systematic Reviews and Meta-Analyses
PSR: Partial Sensory Rhizotomy
TN: Trigeminal Neuralgia
RDC-TMD: Research Diagnostic Criteria for Temporomandibular Disorders
SAS: Self-Rating Anxiety Scale
SDS: Self-Rating Depression Scale
SRS: Stereotactic Radiosurgery
